# FMN-dependent oligomerization of putative lactate oxidase from *Pediococcus acidilactici*

**DOI:** 10.1371/journal.pone.0223870

**Published:** 2020-02-24

**Authors:** Yashwanth Ashok, Mirko M. Maksimainen, Tuija Kallio, Pekka Kilpeläinen, Lari Lehtiö

**Affiliations:** 1 Faculty of Biochemistry and Molecular Medicine, University of Oulu, Oulu, Finland; 2 Unit of Measurement Technology, Kajaani University Consortium, University of Oulu, Kajaani, Finland; University of Queensland, AUSTRALIA

## Abstract

Lactate oxidases belong to a group of FMN-dependent enzymes and they catalyze a conversion of lactate to pyruvate with a release of hydrogen peroxide. Hydrogen peroxide is also utilized as a read out in biosensors to quantitate lactate levels in biological samples. *Aerococcus viridans* lactate oxidase is the best characterized lactate oxidase and our knowledge of lactate oxidases relies largely to studies conducted with that particular enzyme. *Pediococcus acidilactici* lactate oxidase is also commercially available for e.g. lactate measurements, but this enzyme has not been characterized in detail before. Here we report structural characterization of the recombinant enzyme and its co-factor dependent oligomerization. The crystal structures revealed two distinct conformations in the loop closing the active site, consistent with previous biochemical studies implicating the role of loop in catalysis. Despite the structural conservation of active site residues, we were not able to detect either oxidase or monooxygenase activity when L-lactate was used as a substrate. *Pediococcus acidilactici* lactate oxidase is therefore an example of a misannotation of an FMN-dependent enzyme, which catalyzes likely a so far unknown oxidation reaction.

## Introduction

Alpha-hydroxy acids are oxidized by a family of FMN-dependent enzymes [1]. These enzymes use molecular oxygen to convert their substrate to product with the liberation of H_2_O_2_ as a by-product. Members of the enzyme family include lactate oxidase, lactate monooxygenase, flavocytochrome b2 and glycolate oxidase and they are valuable enzymes in biotechnology and in medicine. *Aerococcus viridans* lactate oxidase (AvLCTO) is a prototypical example of lactate oxidase catalyzing the conversion of lactate to pyruvate and liberating H_2_O_2_ as a by-product. It has been used to develop biosensors to measure lactate concentrations in various samples indirectly using H_2_O_2_ [2,3]. In mammals, peroxisomal glycolate oxidase is responsible for conversion of glycolate to glyoxylate, eventually leading to a build-up of oxalate leading to kidney disease and therefore making glycolate oxidase a target for treatment of hyperoxaluria [4].

AvLCTO is thought to catalyze conversion of L-lactate to pyruvate by two half reactions. In the reductive part of the half reaction, FMN is reduced by the substrate and in the oxidative part of the reaction, the enzyme is reoxidized by molecular oxygen. Hydride transfer mechanism proposed based on structural and kinetic studies is now the widely recognized model lactate oxidases. The mechanism involves deprotonation of the substrate’s α-hydroxyl group by His265 and transfer of the α-carbon hydrogen to N5 atom of FMN as a hydride [5–8]. Lactate monooxygenases differ from lactate oxidases only in their oxidative half reaction. Lactate monooxygenases undergo ‘coupled’ reaction wherein the α-keto acid has a longer residence time in the active site, allowing reaction with hydrogen peroxide to undergo oxidative decarboxylation of pyruvate to produce acetate, carbon dioxide and water. This contrasts with lactate oxidases that undergo ‘uncoupled’ pathway, where pyruvate is dissociated immediately before H_2_O_2_ reacts with pyruvate.[9,10]

Previous structural studies of AvLCTO bound to pyruvate revealed the positioning of the active site residues. Tyr40, Arg181, His265, Arg268 interact with the carboxylate group of pyruvate while Tyr146, Tyr215 and His265 interact with keto group [11]. Tyr215 has been shown to be a gatekeeper residue controlling substrate entry and product release. This is achieved by conformational dynamics of the loop. Substitutions in this residue were shown to reduce the rate of product release from the enzyme [12].

Both AvLCTO and *Pediococcus acidilactici* lactate oxidase (PaLCTO) are commercially available enzymes purified from native sources and used for biosensor applications [13]. While AvLCTO has been characterized extensively using recombinant system, PaLCTO has not been studied. PaLCTO is annotated as lactate oxidase and its closest homolog in *Pediococcus acidilactici* proteome does not use lactate as a substrate. We describe here structural and biophysical studies of PaLCTO, which revealed FMN-dependent folding and oligomerization of the enzyme.

## Materials and methods

### Cloning and site directed mutagenesis

*Pediococcus acidilactici* proteome wide search for keywords lactate, lactate oxidase, α-hydroxy acid in Uniprot database gave only one hit that matched with AvLCTO. Similarly, BLASTp search with AvLCTO against *P*. *acidilactici* DSM20284 revealed the same hit annotated as putative L-lactate oxidase (Uniprot E0NE46). Genomic DNA of *Pediococcus acidilactici* (DSM 20284) and *Aerococcus viridans* (DSM 20340) was obtained from DSMZ, Germany. Gene encoding PaLCTO was cloned into pNH-TrxT (Structural Genomics Consortium) vector using SLIC cloning. The vector encodes for an N-terminal thioredoxin tag with a cleavable TEV protease recognition site. A94G mutant was obtained using standard site-directed mutagenesis protocol. All clones were verified using Sanger’s dideoxy sequencing. Untagged PaLCTO and AvLCTO were recombinantly expressed from pNIC-CH vector with a stop codon added immediately after the native sequence. Commercial PaLCTO and AvLCTO were purchased from Sigma (catalog number for PaLCTO LO638, lots STBG2905V and STBF3223V; and for AcLCTO L9795).

### Protein expression and purification

Proteins were expressed using BL21 (DE3). Overnight culture was inoculated into terrific broth auto-induction media supplemented with 8 g/L glycerol and 50 μg/ml kanamycin. Cells were grown at 37°C until OD_600_ reached 1. The temperature was reduced to 18°C for overnight for protein expression. Cells were harvested the next day by centrifugation and either directly used for purification or flash frozen.

Cells were resuspended in lysis buffer (50 mM Hepes pH 7.5, 0.5 M NaCl, 10% glycerol, 0.5 mM TCEP, 10 mM imidazole) with 0.1 mM Pefabloc SC (Sigma-Aldrich) and lysed at 20 kPsi using cell disruptor (Constant systems, UK). Lysates were cleared by centrifugation at 16,000 rpm for 30 minutes. Supernatant was loaded on 4 ml of HisPur^TM^ Ni-NTA resin (Thermo Scientific). The beads were washed with 10 ml of lysis buffer and then with 15 ml of wash buffer (50 mM Hepes pH 7.5, 0.5 M NaCl, 10% glycerol, 0.5 mM TCEP, 25 mM imidazole) was used to capture recombinant proteins. Proteins were eluted with elution buffer (50 mM Hepes pH 7.5, 0.5 M NaCl, 10% glycerol, 0.5 mM TCEP, 350 mM imidazole). Buffer exchange was achieved using Amicon 30 kDa concentrator and the tags were cleaved by digestion with TEV protease (in-house preparation) for 2 days at 4°C [14]. Supernatant was passed again through Ni-NTA resin to capture thioredoxin and TEV protease. Tag cleaved PaLCTO was collected from flow through. The proteins were purified subsequently with HiLoad 16/600 Superdex 200 using size exclusion buffer (30 mM Hepes pH 7.5, 0.35 M NaCl, 10% glycerol, 0.5 mM TCEP). Proteins were concentrated and were flash frozen in liquid N_2_ and stored at -70°C. Purified PaLCTO showed bright yellow color due to the presence of a co-factor.

For purification of untagged PaLCTO and AvLCTO, cells were resuspended in 20 mM Potassium Phosphate pH 7.0 with 0.1 mM Pefabloc and lysed as described above. Clarified lysate was loaded into Q sepharose, followed by washing with lysis buffer. Proteins were eluted using 0–1 M KCl gradient in lysis buffer. Fractions containing required proteins were pooled and solid ammonium sulfate was added to a concentration of 1.5 M. After 15-minute incubation on ice precipitates were discarded using centrifugation. Proteins were loaded to phenyl sepharose and washed with 20 mM Potassium phosphate, 1.5 M ammonium sulfate and were eluted using a gradient from 1.5 M ammonium sulfate to 0 M in 20 mM Potassium Phosphate pH 7.0. Pooled from phenyl sepharose were loaded to Superdex200 16/600 equilibrated with 100 mM Potassium phosphate pH 7.0.

### Analytical size exclusion chromatography (aSEC)

Bio-Rad gel filtration standards were used for calibrating Superdex 200 Increase 10/300 column. 30 mM Hepes pH 7.5, 350 mM NaCl, 0.5 mM TCEP was used as buffer with 0.5 ml/min flow rate. Blue dextran was used for void volume calculation. Protein elution volumes were calculated using peak elution time with absorption at 280 nm. Partition coefficient (K_av_) were calculated using elution volumes with the equation K_av_ = (V_e_-V_0_)/ (V_t_-V_0_), where V_e_ is the elution volume, V_0_ is the void volume and V_t_ is the total volume of column.

### Deflavination and reflavination

Protein aliquots were thawed and diluted in 250 mM sodium phosphate pH 7.5, 3 M Potassium bromide, 10% glycerol, 0.5 mM TCEP, 1 mM EDTA and left on ice for 4 days [15]. Following incubation, solution was concentrated using a 10 kDa cut-off concentrator. Majority of the yellow color was in the flow through. Deflavination was repeated to completely remove FMN and the yellow color. Pure monomeric proteins were obtained using size exclusion chromatography using size exclusion buffer.

For FMN incorporation experiments, 1:2 molar ratio (protein:FMN) was used and incubated on ice for overnight and subsequently purified using SEC in Superdex200 16/600 using 30 mM Hepes pH 7.5, 0.35 M NaCl, 10% glycerol, 0.5 mM TCEP.

### CD spectroscopy

CD spectra were recorded using Chirascan^TM^ spectrophotometer (Applied photophysics Ltd, USA). Spectra scan experiments were conducted at 22°C using a 0.1 cm optical path length cuvette. Proteins were diluted to 0.1 mg/ml in water for measurements. Spectra were recorded from 190–280 nm in 1 nm steps thrice and averaged for each sample. Thermal stability measurements were done by heating the protein 22–80°C. Data analysis was carried out by using pro-data viewer (Applied Photophysics Ltd, USA). Data obtained at 222 nm was used for calculating melting temperature using Boltzmann sigmoidal equation in Graphpad Prism.

### Crystallization

Crystals were readily obtained in several conditions from the initial crystallization trials using the JCSG+ and PACT premier^TM^ crystallization screens (Molecular Dimensions). Crystals used for data collection were obtained in 24 hours either in a hanging or sitting drops, which were prepared by mixing 75–150 nl of 15 mg/ml protein with 75–150 nl of 1.1 M Sodium Malonate, 0.1 M Hepes pH 7.0, 0.5% Jeffamine ED-2003 at 20°C.

### Data collection, processing & refinement

The crystals were cryoprotected with a solution containing 20% (v/v) glycerol in the mother liquor and flash-frozen in liquid nitrogen. All measurements were carried out at 100 K. WT PaLCTO dataset was collected at ID30A-1 and refolded dataset was collected at ID30B (European Synchrotron Radiation, France). Mutant dataset was collected at I04 (Diamond Light Source, UK). Diffraction data were indexed, scaled and merged using XDS [16]. Phases for the wild type PaLCTO were solved using molecular replacement with Phaser MR of the Phenix package [17,18] using AvLCTO (2J6X) as a search model [19]. Iterative model refinement was carried out using Refmac5 in CCP4 [20]. Model building and visualization were performed using Coot [21,22]. Structure related figures were generated using Pymol (Schrödinger). The data collection and structure refinement statistics are presented in Table 1.

**Table 1:** Data collection and refinement statistics for the crystal structures.

Structure	Native WT PaLCTO	A94G PaLCTO	Refolded WT PaLCTO
PDB code	6RHT	6R9V	6RHS
Beamline	MASSIF 1, ESRF	I04, DLS	ID30B, ESRF
Wavelength (Å)	0.9660	0.9795	0.9677
Space group	I422	I422	I422
Cell dimensions a, b, c (Å)	135.24 135.24 124.84 90.0 90.0 90.0	135.53 135.53 124.86 90.0 90.0 90.0	135.50 135.50 125.69 90.0 90.0 90.0
Resolution (Å)	95.6–1.9	50–2.0	50–2.6
R_merge_	16.0 (114)	19.5 (123.3)	21.9 (131.6)
I / σI	10.3 (2.2)	11.98 (2.17)	11.70 (2.19)
CC 1/2	99.6 (70.4)	99.7 (79.8)	99.8 (75.5)
Completeness (%)	99.9 (100)	100 (100)	100 (100)
Redundancy	8.9 (9.1)	13.2 (13.6)	13.3 (13.3)
**Refinement**			
R_work_ / R_free_	0.182 / 0.211	0.148 / 0.173	0.186/ 0.216
**No. atoms**			
Protein	2895	2800	2743
Ligand FMN	31	31	31
Water	124	236	20
**B-factors**			
Protein	29.62	25.25	40.3
Ligand FMN	23.2	21.74	31.3
Glycerol	52.05	48.64	54.77
Water	30.96	34.95	32.34
**R.m.s.d.**			
Bond lengths (Å)	0.010	0.009	0.009
Bond angles (°)	1.628	1.54	1.675
**Ramachandran plot (%)**			
Favored regions	96.69	97.28	96.34
Additionally allowed regions	3.04	2.45	3.38
outliers	0.28	0.27	0.28

### Small angle X-ray scattering

SAXS was conducted in size-exclusion chromatography mode. Scattering contributions due to buffer were subtracted from protein peak using ScÅtter. SAXS based molecular weight estimates were done using SAXS MoW 2.0 [23]. Analysis of crystal structure fitting to scattering profiles was done using CRYSOL [24,25].

### Activity assays

All experiments were conducted in standard atmospheric pressure and at 37°C. Oxidase activity towards L-lactate and glycolate was measured in reaction mixture containing aminoantipyrine (4-AAP) and N,N-dimethylaniline (DMA) that react to form quinonediimine dye when they are oxidized by the horseradish peroxidase enzyme (HRP) and hydrogen peroxide generated in lactate oxidase reaction [9]. The initial reaction cocktail contained for each reaction 40 μl of 200 mM 3,3 dimethylglutaric acid-NaOH buffer, pH 6.5 (DMGA), 20 μl HRP solution (50 U/ml, Sigma-Aldrich P-8250), 20 μl 4-AAP and 30 μl deionized ELGA water. The cocktail was mixed by inversion and 110 μl was pipetted into microplate wells in quadruples. Next, 25 μl of substrate solution and 40 μl DMA 0.2% (v/v) solution was added into the wells. The microwell plate was transferred to a reader (Varioskan Flash or Tecan infinite M1000 pro) and incubated at +37°C for 5 min. Finally, 25 μl of enzyme dilution (~ 2 μg/ml) in 10 mM sodium phosphate buffer containing 0.1 mM flavin mononucleotide co-factor (FMN, Sigma-Aldrich F2253) was dispensed into the wells and absorbance at 565nm was monitored with one minute intervals for 15 minutes. Oxidase activity was calculated by dividing the slope of the linear regression line of enzyme activity graph (A565nm) from t = 0 to t = 15 min by the amount of enzyme protein taken to the assay. Standard L-lactate concentration in the assay was 1 mM, but when enzyme activities were screened, concentrations used ranged from 1 mM to 62.5 mM, and protein amount in the assay was increase even 250-fold. In K_m_ determinations, L-lactate concentrations were from 0.2 mM to 2.0 mM. Instead of FMN, flavin adenine dinucleotide (FAD) was tested as a co-factor in some enzyme assays.

For lactate 2-monooxygenase assay, protein samples were incubated in a similar mixture as previously, but 4-AAP and DMA were omitted. In addition to Na-phosphate buffer, 10 mM imidazole-HCl, 150 mM NaCl (pH 7.0) and 30 mM Hepes, 150 mM NaCl (pH 7.0) buffers were tested, since phosphate inhibits some enzymes using lactate as a substrate. All three buffers contained also 10 mM FMN. The assays were incubated overnight at +37°C. Active lactate oxidases were used as controls to demonstrate detection and quantitation of activity. After incubation, reaction mixtures were analyzed by capillary electrophoresis. The mixtures were filtered with a 0.45 μm GHP Acrodisc syringe filter prior to analysis that were carried out with a P/ACE MDQ CE instrument (Beckman-Coulter, Fullerton, CA, USA) equipped with a diode array detector (DAD) using uncoated fused-silica capillaries of I.D. 25 μm and length 30/40 cm (effective length/total length). The samples were injected at a pressure of 3447.4 Pa for 10 s with a separation voltage of +16000 V. Calibration curves were created for external quantification. In addition, sample runs were performed also with spiked standards to confirm the identity of the analytes. Similar capillary electrophoresis assay was used also to test oxidase activity of PaLCTO towards 4-hydroxy mandelate.

## Results

### Characterization of purified protein

**Fig 1:**
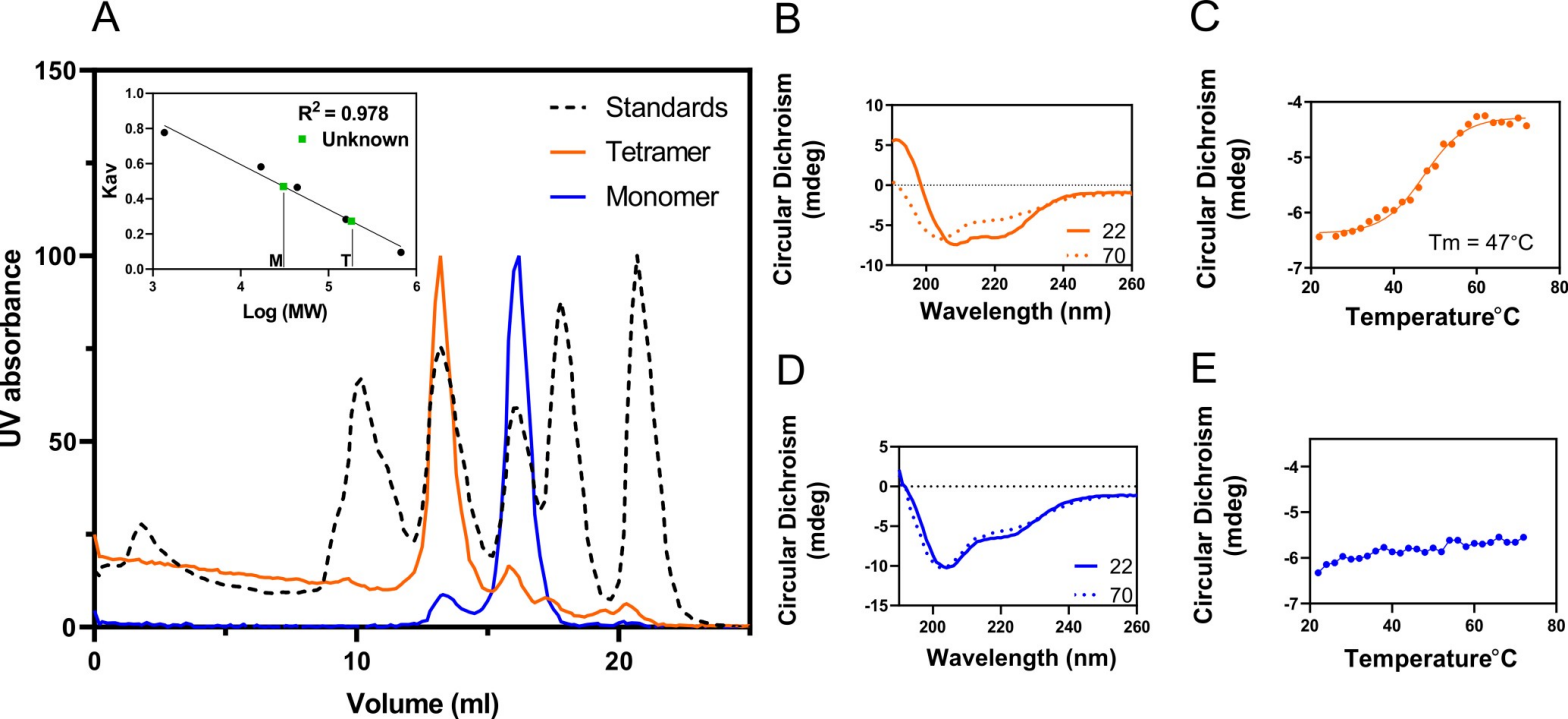
Characterization of purified PaLCTO. a) analytical size exclusion chromatography of purified tetrameric and monomeric fractions of PaLCTO. (inset: calibration curve. M- Monomer T- tetramer). b) CD spectra of tetrameric PaLCTO at 22 and 70°C. c) Melting temperature analysis of tetrameric PaLCTO at 222 nm. d) CD spectra of monomeric PaLCTO at 22 and 70°C. e) Melting temperature analysis of monomeric PaLCTO at 222 nm.

Recombinant enzyme was produced in *E*. *coli* and purified using Ni^2+^-NTA affinity chromatography. During subsequent size exclusion chromatography, PaLCTO eluted as two peaks. The first peak was yellow in color and the second was colorless (S1 Fig). Mass spectrometric analysis of the proteins present in the fractions revealed that both peaks were PaLCTO. Previous reports demonstrate that the other members of this enzyme family are oligomeric and in order to study the oligomerization properties of the protein in both fractions, analytical size exclusion chromatography was performed with molecular weight standards (Fig 1A). The first peak containing the yellow fraction was judged to be tetrameric with a molecular weight of 162 kDa, while the colorless protein was monomeric with a molecular weight of 33 kDa. These results are in good agreement with the theoretical molecular weight of a monomer (39 kDa) and of a FMN containing tetramer (158 kDa).

Flavin mononucleotide (FMN) is the co-factor that imparts bright yellow color to the protein and since this is absent in the monomeric protein, circular dichroism (CD) spectroscopy was performed to assess any possible differences in secondary structure between tetrameric and monomeric proteins. Tetrameric fraction showed typical features of a folded protein rich in secondary structures (Fig 1B). Negative peak maxima at 202 and 222 nm and a positive peak at 195 nm indicated that the protein has an alpha-helical content. Monomeric protein showed negative peak maxima at 204 nm, indicating that the monomeric protein showed no typical characteristics of a folded protein (Fig 1D). Melting curve studies using CD were performed to determine if the proteins are folded. Tetrameric fraction clearly showed sigmoidal transition at 222 nm whereas monomeric fraction did not show any such transition (Fig 1C and 1E). The calculated melting temperature of the tetrameric protein was 47 ± 0.4°C. These results indicated that the monomeric protein has little or no secondary structural elements typical for a folded protein.

**Fig 2:**
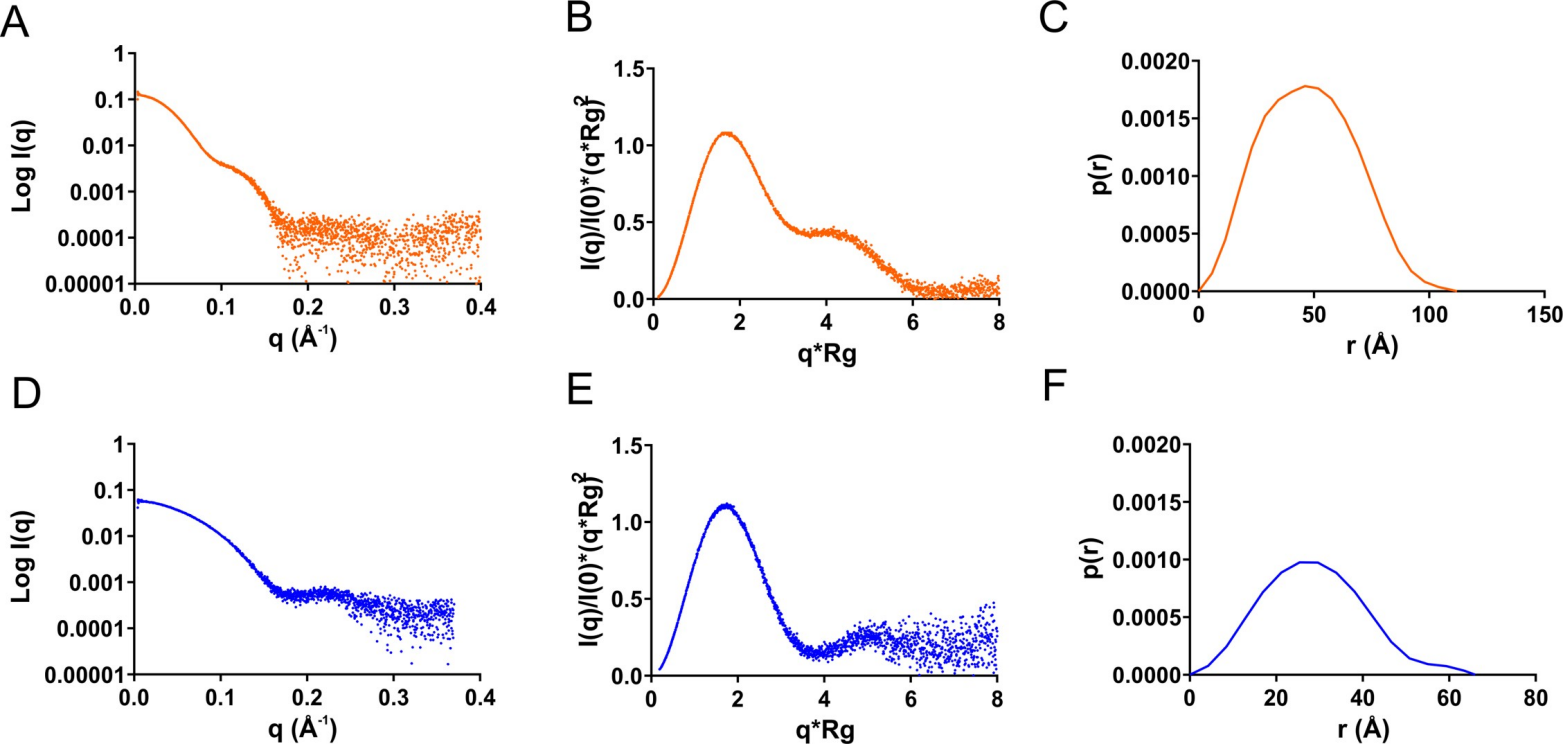
SAXS analysis of tetrameric and monomeric PaLCTO. a) Experimental scattering profile b) Normalized Kratky plot and c) P(r) distribution curves for tetrameric native PaLCTO. d) Experimental scattering profile e) Normalized Kratky plot and f) P(r) distribution curves for monomeric PaLCTO.

We also performed small angle X-ray scattering (SAXS) analysis for both of the proteins to further strengthen data obtained from CD and aSEC (Fig 2A and 2D). Analysis of raw scattering curves from tetrameric and monomeric fractions with SAXS MOW 2.0 indicated that the proteins had a molecular weight of 153 and 40 kDa, respectively. Normalized Kratky plot analysis revealed that both of the proteins were actually globular (Fig 2B and 2E), which was surprising in the light of the CD analysis of the monomer (Fig 1D). For a completely disordered protein one would expect the Kratky plot to show a constant rising in increased angle and therefore it appears that the monomer stays in a globular form despite that it does not contain distinct secondary structures. Distance distribution analysis shows Dmax values for the tetrameric and monomeric protein to be 113 Å and 66 Å, respectively (Fig 2C and 2F) and the tetramer Dmax correlates well with the crystal structure of AvLCTO. A summary of SAXS parameters obtained is summarized in (S1 Table and S2 Fig).

### Co-factor dependent oligomerization

**Fig 3:**
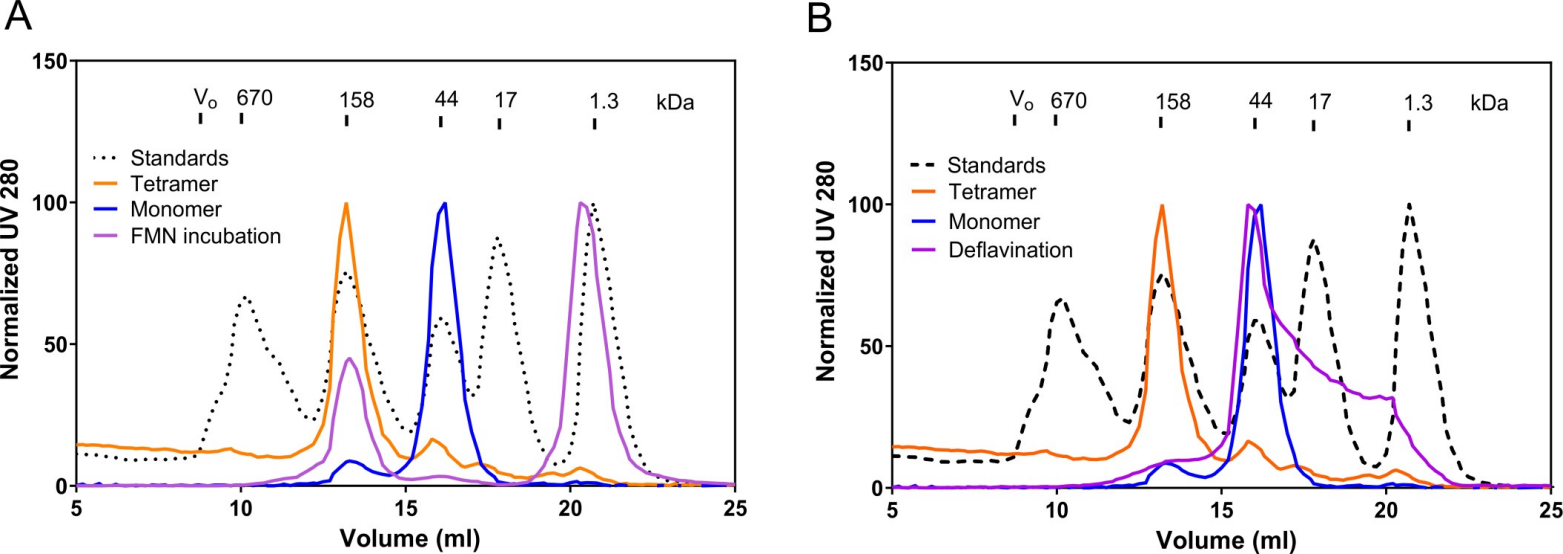
FMN dependent oligomerization of PaLCTO. a) Monomeric PaLCTO upon incubation with FMN forms a tetramer that elutes at the same elution volume as native PaLCTO. b) Deflavination of native PaLCTO makes it monomeric causing it to elute in monomer elution volume.

In order to study whether monomeric enzyme could incorporate the co-factor, we incubated it with FMN and subsequently carried out SEC analysis to see if the protein oligomerizes in the presence of the co-factor. The protein indeed eluted at the same elution volume as native tetrameric protein (Fig 3A) indicating co-factor dependent oligomerization. Previously this phenomena has been described for some FAD containing enzymes [26,15,27]. We also performed loss of FMN binding studies, where native tetrameric proteins were chemically treated to remove FMN and the deflavination resulted in monomeric proteins (Fig 3B).

**Fig 4:**
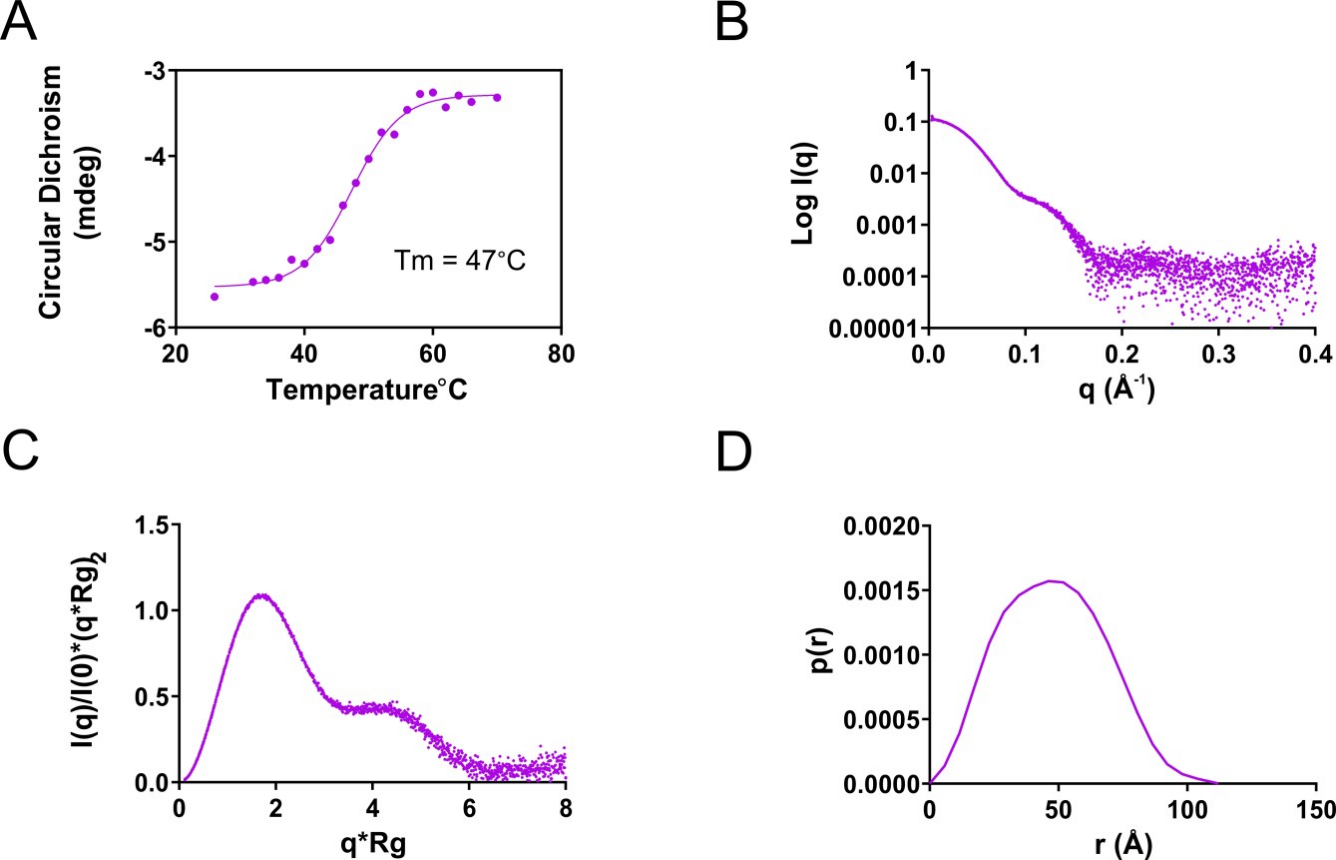
Analysis of refolded PaLCTO. a) Melting temperature analysis of refolded PaLCTO using CD from 222 nm. b) Experimental scattering profile. c) Normalized Kratky plot of refolded enzyme. d) P(r) distribution of refolded PaLCTO.

To assess if refolded proteins have similar secondary structure to native PaLCTO, we performed CD spectroscopic studies. Refolded protein had similar spectra to WT native PaLCTO and melting temperature (47 ± 1°C) (S3A Fig and Fig 4A). Together the results show that PaLCTO could be reversibly folded and unfolded *in vitro*. SAXS analysis of refolded PaLCTO (Fig 4B and 4C) were also in line and indicated that the protein had a molecular weight of 151 kDa, Kratky plot was similar to native PaLCTO (Fig 4C) and Dmax was 113 Å as for the native tetramer (Fig 4D and S1 Table).

Recombinant AvLCTO showed only one peak in SEC corresponding to tetrameric protein (data not shown) and deflavination for AvLCTO using same protocols used for PaLCTO was not successful. Other methods such as urea induced denaturation resulted in protein aggregates upon urea removal, precluding similar studies with AvLCTO. Consistent with the above mentioned observations, the melting temperature of AvLCTO was higher (T_m_ = 59 ± 1°C) compared to PaLCTO (T_m_ = 47 ± 1°C) with a ΔT_m_ of 12°C (S3 Fig). These results show that AvLCTO has a much more stable quaternary structure than PaLCTO and the co-factor dependent assembly of enzyme seems to be a specific property of PaLCTO.

### Crystal structure of WT PaLCTO

**Fig 5:**
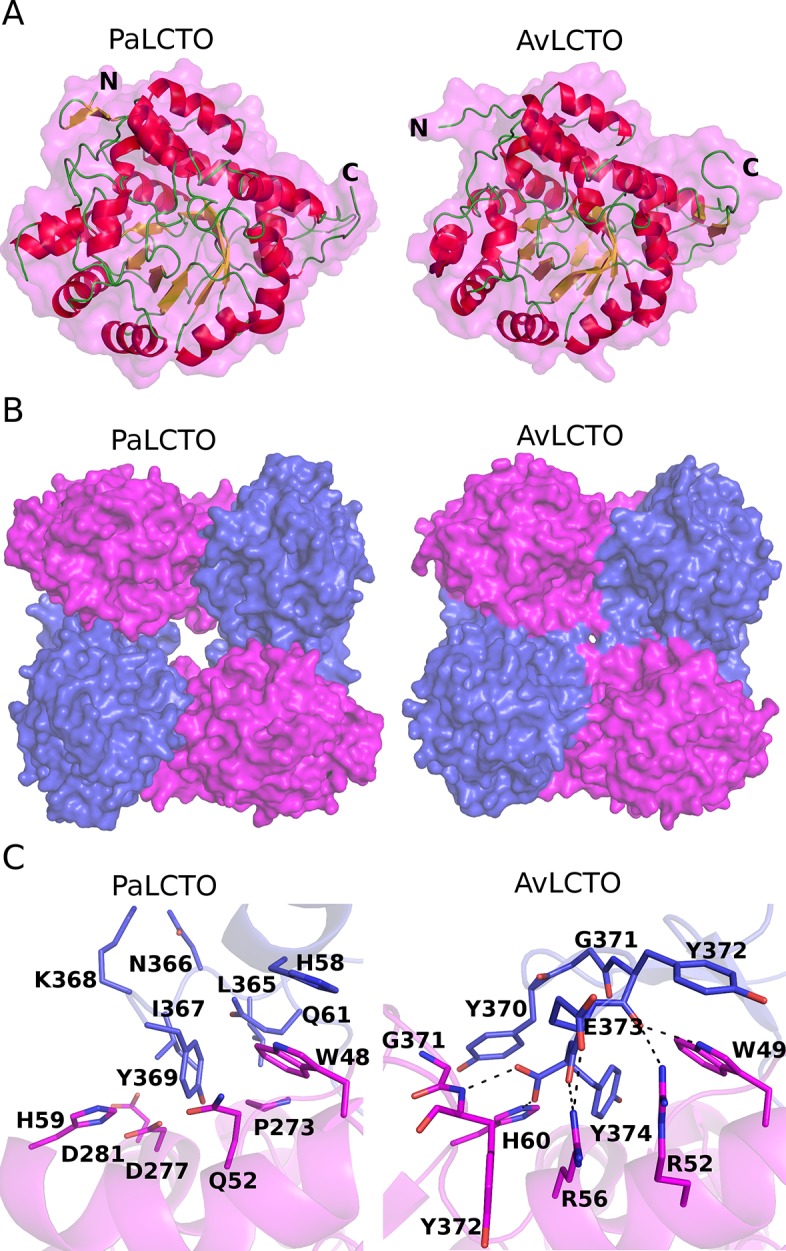
Crystal structure of PaLCTO and structural comparison of PaLCTO and avLCTO tetramers. a) PaLCTO and avLCTO monomers are presented as ribbon-surface models. Beta-strands and alpha-helixes are colored in yellow and red, respectively. Loops and protein surface are colored in green and magenta, respectively. The N- and C-terminus are indicated with the corresponding letters b) PaLCTO and avLCTO tetramers are presented as surface models. Protein molecules are colored in magenta and blue in turn. c) The interface areas of avLCTO and PaLCTO monomers. The residues are presented as cartoon and stick models and also colored as in B. Hydrogen bond interactions are indicated with black dash lines.

We crystallized PaLCTO (S4 Fig) and solved it’s structure at 1.9 Å resolution (Table 1). Refinement statistics are good and the residue highlighted as a Ramachandran outlier, Ser296, has good electron density and is interacting closely with the co-factor explaining its unfavorable conformation. The asymmetric unit contains one molecule and the monomer has a typical TIM barrel structure containing eight alpha- helices and beta-strands. Superimposition of WT PaLCTO and AvLCTO monomer shows that the structures are highly similar (rmsd = 0.70 Å for 305 C-alpha atoms). The main structural differences between the monomers of WT PaLCTO and AvLCTO can be observed in the N- and C-termini (Fig 5A). Firstly, there is an additional β-hairpin composed by the residues 2–9 in the N-terminus of PaLCTO. Secondly, the C-terminus of PaLCTO is three residues shorter compared to AvLCTO.

We then analyzed the structure with Pisa server which gave a biological assembly likely similar to AvLCTO. These results are consistent with other data presented above that the protein is tetrameric in solution. Analysis of PaLCTO tetramer with SAXS data measured in solution had a chi^2^ of 1.6 (S5 Fig) which gives additional confirmation that the protein is a tetramer. Structural comparison of the PaLCTO and AvLCTO tetramers shows a similar assembly but it also reveals that the PaLCTO tetramer has a much larger central cavity than AvLCTO due to the shorter C-terminus (Fig 5B). The buried surface area between monomers of AvLCTO is larger (1737 Å^2^) when compared to (1452 Å^2^) from PaLCTO. There are 22 hydrogen bond interactions including 2 salt bridges in the monomer-monomer interface area of PaLCTO, whereas the AvLCTO monomer-monomer interface area contains 28 hydrogen bonds including 7 salt bridges. The C-terminal residues (Tyr372, Glu373 and Tyr374) of AvLCTO are forming almost a third of the interactions as they are forming 8 of the hydrogen bonds and two salt bridges. The C-terminus of AvLCTO contributes to the oligomer formation through a network of ionic interactions (C-terminus, Glu373, Arg52, Arg56), through hydrophobic contact and multiple hydrogen bonds (Fig 5C). The sidechain of Tyr370 stacks with the imidazole ring of His60 from the neighboring subunit and makes the central cavity smaller compared to PaLCTO (Fig 5C). These structural features provide a rationale as to why AvLCTO has higher stability than PaLCTO.

### The active site of WT PaLCTO and two conformations of the active site loop

**Fig 6:**
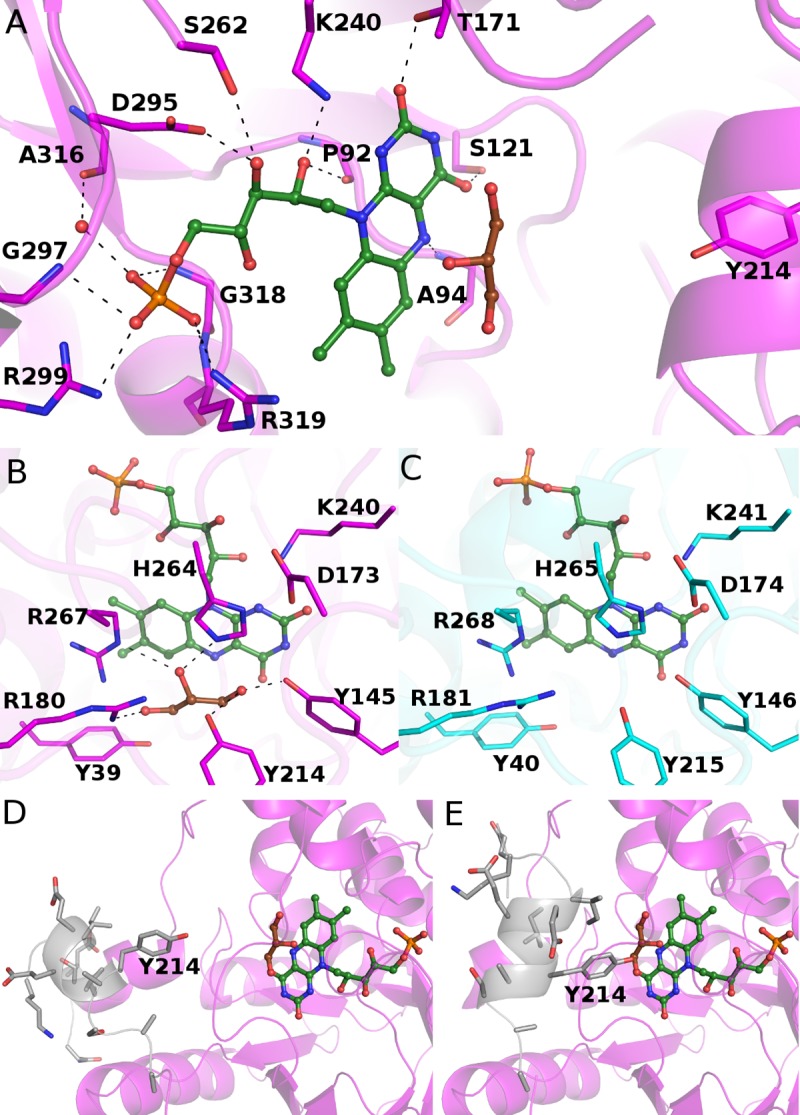
Binding of FMN in WT PaLCTO. a) Hydrogen bonding network between flavin and WT PaLCTO. Flavin and glycerol are presented as stick-sphere models and colored in green and brown, respectively. The residues of WT PaLCTO are presented as cartoon-stick models and colored in magenta. The hydrogen bonds between the flavin and residues are indicated with black dash lines b) Active site of PaLCTO colored as in A c) Active site of AvLCTO. The residues are colored in cyan d) Open form WT PaLCTO. The main polypeptide chain having two conformations is colored in grey and presented as ribbon-stick model. Tyr214 is indicated with a label. e) Closed form of WT PaLCTO. The main polypeptide chain and Tyr214 are presented as in d.

The hydrogen bonding network between FMN and the surrounding residues contains in total twelve interactions (Fig 6A). 2Fo-Fc electron density map for FMN is shown in S6A Fig. We also observed a glycerol molecule from the cryoprotectant solution bound close to FMN in an expected substrate binding site, where pyruvate (product of the enzyme) has been modelled [11]. Comparison shows that the active site residues of PaLCTO and AvLCTO are highly conserved. All the residues surrounding the co-factor are same and in similar conformation (Fig 6B and 6C) [11,19].

Crystal structure of AvLCTO in complex with the lactate oxidase product pyruvate has been determined earlier [11] and it has been described that the key residues for catalysis are Tyr40, Tyr215, His265 and Arg268. A conserved catalytic triad of (Tyr40, Tyr146, Arg268) positions L-lactate in the active site, Tyr40 and Arg268 interact with the carboxyl group of the substrate, and Tyr146 and Tyr215 interact with the keto group of the lactate C2 carbon. The glycerol observed in the PaLCTO active site mimics the substrate and interacts with these residues (Tyr145, Arg180, Tyr214, His264 and Arg267).

Recent experimental evidence has shown that Tyr215 controls entry and exit of substrate and product in AvLCTO as mutations in Tyr215 result in a change in time of product release [12]. The corresponding residue in PaLCTO is Tyr214 and due to similarity, the loop could have a similar role in this enzyme. Interestingly, we observed that the main polypeptide chain between Ser201 –Ala219 of PaLCTO (loop 4) exists in two conformations (Fig 6D and 6E) in the crystal structure. Because of lack of electron density, we were not able to build both conformations completely and therefore the open conformation lacks the residues Gly205-Gly207 and the closed conformation lacks the residues Ser201-Asn204 in the model. Despite the incomplete structure of the chain, the role of the region could be seen as controlling substrate entry and product release by opening and closing the active site of PaLCTO. In the closed conformation, the OH group of Tyr214 comes very close to the expected substrate having 2.6 Å distance to the O1 atom of the glycerol (Fig 6E). Instead in the open conformation, the distance between the OH group and the O1 atom is 7.5 Å (Fig 6A and 6D).

### Structure of PaLCTO mutant A94G

We generated a mutant (A94G) based on literature precedent suggesting that the mutation of the residue may convert the enzyme to accept also larger substrates [28]. The mutant was crystallized in the identical conditions as WT PaLCTO and the structure was determined at 2.0 Å resolution. The superimposition of the mutant and WT monomer structures showed high structural identity (rmsd = 0.16 Å for C-alpha 337 atoms). However, in the mutant structure we only observed the open conformation for the main polypeptide chain (loop 4: S201 –A219) and we were able to build the conformation completely with all the residues. 2Fo-Fc electron density map of loop 4 (Ser201-Ala 219) is shown in S6B Fig. The open conformation of PaLCTO A94G varies compared to the AvLCTO variant A95G (PDB: 4RJE) where a closed conformation for the corresponding loop 4 is observed [12]. Mutation of A94G likely reduces loop dynamics and therefore is completely resolved in the structure. The structures lay the ground for future biochemical experiments to determine the impact of mutation on loop dynamics. A stereo view of the A94G mutant aligned to WT PaLCTO is shown in (S7B Fig).

### Structure of refolded PaLCTO

Since refolded PaLCTO behaved similarly in above described experiments, we subjected refolded PaLCTO to crystallization trials and obtained crystals in conditions described for WT native enzyme. The structure was solved to 2.60 Å resolution. A stereo view of refolded PaLCTO aligned to WT native PaLCTO is shown in (S7A Fig). The structure proved that PaLCTO gains the same three-dimensional structure as WT PaLCTO when refolded. We also saw signs of two conformations for the chain S201 –A219 in the structure of refolded PaLCTO, but due to modest resolution and lack of electron density the chains could not be built. Like WT enzyme the refolded oligomer fitted well to the SAXS data (S5B Fig).

### PaLCTO appears not to be a L-lactate oxidase

We used recombinant in-house produced AvLCTO to benchmark against commercially available AvLCTO. The produced enzyme was active displaying nearly identical activity and a K_m_ value (0.52 ± 0.05 mM) compared to commercial AvLCTO (0.52 ± 0.06 mM) (S8 Fig). V_max_ values were 2900 units/min/mg and 3400 units/min/mg, respectively. With the above-mentioned controls tested with the assay, we performed activity assays for PaLCTO using L-lactate as a substrate. To our surprise we were unable to obtain any activity even with 62.5 mM of L-lactate (~100-fold higher than K_m_ of AvLCTO) and enzyme concentrations up to 250-fold to those used for AvLCTO assay. Initially we used PaLCTO which was produced with a His tag that was subsequently removed with a protease for activity assays and further tests with PaLCTO that was expressed and purified without any tags gave identical results without any activity. These results were puzzling and therefore a commercially available *Pediococcus sp*. lactate oxidase was used as a control and interestingly, commercial *Pediococcus sp* lactate oxidase was active in our assays. To explain this observation, we performed SDS-PAGE of commercial enzyme. Bands were excised and analyzed using mass spectrometry and the commercial PaLCTO was in fact identified as AvLCTO. This was further confirmed by analyzing an additional lot of the commercial enzyme with same results. We used in-house produced AvLCTO and PaLCTO as controls for mass spectrometry to see if it could identify them and they were distinguished correctly. It is likely that a mistake had been made long time ago when the microbial species was identified and has persisted ever since from commercial sources. These results indicate that commercially sold enzyme at least from this specific commercial source is AvLCTO. Additionally, we also tested other substrates such as glycolate and 4-hydroxy mandelate which however gave no activity.

Lactate 2-mono-oxygenase is an enzyme with very similar structure and reaction mechanism to lactate oxidase [10]. Therefore, we assayed also lactate 2-mono-oxygenase activity of the in-house produced PaLCTO. These results were negative as there was no acetic acid formed from L-lactate, and neither did it react into any other product, since the concentration of L-lactate was unchanged in the reaction mixture (S9 Fig and data not shown).

## Discussion

We started with the aim of characterizing recombinant PaLCTO enzyme. Although successful in producing the protein and determining the structure, we were not able to observe enzymatic activity when L-Lactate was used as substrate. Given that these enzymes were produced with affinity purification tag, we wondered if this led to loss of enzymatic activity. We also produced recombinant tag free enzyme which gave no activity. As a control we used in-house produced AvLCTO with both affinity tag (tag cleaved later during purification) and tag free version. Both of these versions of AvLCTO had enzymatic activity similar to earlier reports [12]. We also tested other substrates such as glycolate and 4-hydroxy mandelate with PaLCTO but no activity could be seen. Additional experiments for testing lactate monooxygenase activity also failed to detect any acetate as product or disappearance of L-lactate. While this study was in progress a paper describing structure of lactate monooxygenase from *Mycobacterium smegmatis* (MsLMO) was published [29]. Oxidase and monooxygenase enzymes have similar mechanism (see introduction), but monooxygenase enzymes undergo ‘coupled’ pathway. In *M*. *smegmatis* and other lactate monooxygenases loop 4 is significantly longer and forms a defined folded structure in MsLMO (PDB:6DVI). In MsLMO crystal structures, loop 4 is about 49 residues long and structured elements in the loop have been hypothesized to impede loop 4 dynamics, thereby oxidizing pyruvate further to acetate. Unlike other oxidases including PaLCTO structure, loop 4 in MsLMO is clearly visible, indicating that the loop is less dynamic than in other oxidases. Clearly loop 4 in PaLCTO is more similar to AvLCTO than MsLMO. Given that the active site residues are conserved, and the loop 4 structure is similar to AvLCTO, PaLCTO would catalyze a similar reaction as AvLCTO, although the identity of the substrate is not known. Commercially available PaLCTO turned out to be AvLCTO and further studies are required to definitively specify the origin of the enzyme. These results could indicate that *P*. *acidilactici* does not have any active lactate oxidase but does not rule out from other species. The inactivity of the recombinant enzyme could be in the future be confirmed by testing *P*. *acidilactici* strain lysates grown under different conditions to confirm both expression of PaLCTO and possible lactate oxidase activity.

We report to our knowledge the first protein that can reversibly form oligomers in presence of FMN, while several other examples of FAD containing proteins exhibit this behavior [15,26,27]. This observation was specific to PaLCTO as it was not observed for AvLCTO. Based on structural studies we believe that the C-terminal residues provide inter subunit interactions that stabilize AvLCTO tetramer and the absence of such interactions makes PaLCTO susceptible to changes in oligomeric state. An interesting observation with deflavinated monomeric PaLCTO was that although it lacked any secondary structure elements it was still globular based on SAXS data. One useful feature of PaLCTO, is that there are no cysteines in the wild-type protein. This allows one to introduce site-specific cysteines that could allow cysteine labelling by fluorophores and other small molecules to monitor protein folding. With high expression levels, protocols for folding and unfolding and availability of crystal structure, makes PaLCTO potentially an attractive model protein to study protein folding.

### Accession codes

Atomic coordinates and structure factors have been deposited to Protein Data Bank with accession codes 6RHT (WT), 6RHS (refolded) and 6R9V (A94G mutant).

## Supporting information

S1 Fig SEC chromatogram of PaLCTO. (PNG)

S2 Fig Guinier fits of SAXS data. (PNG)

S3 Fig CD spectra and melting curves of refolded PaLCTO & avLCTO. (PNG)

S4 Fig Crystals of PaLCTO. (PNG)

S5 Fig CRYSOL fitting of SAXS data. (PNG)

S6 Fig 2Fo-Fc map of relevant regions. (PNG)

S7 Fig Stereo view of structures. (PNG)

S8 Fig Comparison of activity produced in-house and commercially available AvLCTO. (PNG)

S9 Fig Lactate monooxygenase activity. (PNG)

S1 Table SAXS summary. (PDF)
