# Supplementary figures and images for: FMN-dependent oligomerization of putative lactate oxidase from Pediococcus acidilactici
Source: PLoS One. 2020 Feb 24;15(2):e0223870. doi: 10.1371/journal.pone.0223870 (PMC7039449; doi:10.1371/journal.pone.0223870)

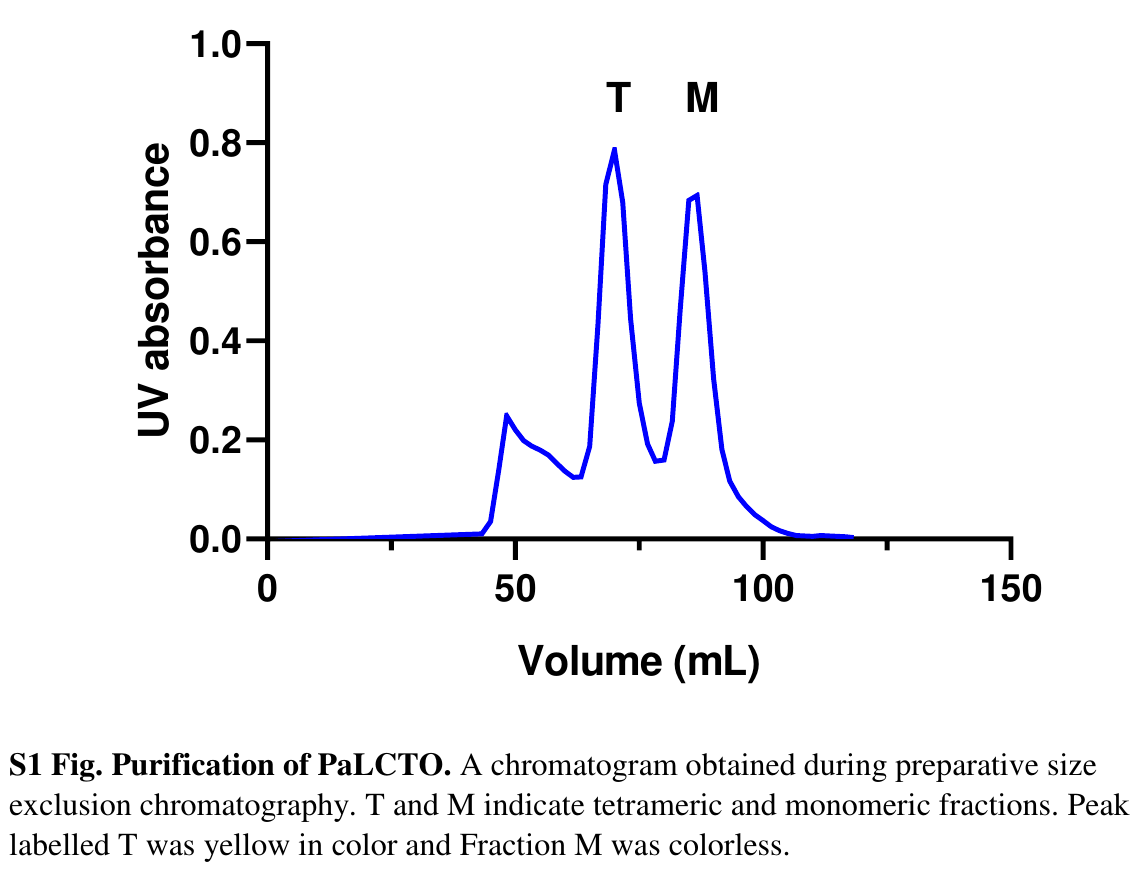

Supplement: S1 Fig — (PNG) [file pone.0223870.s001.png]

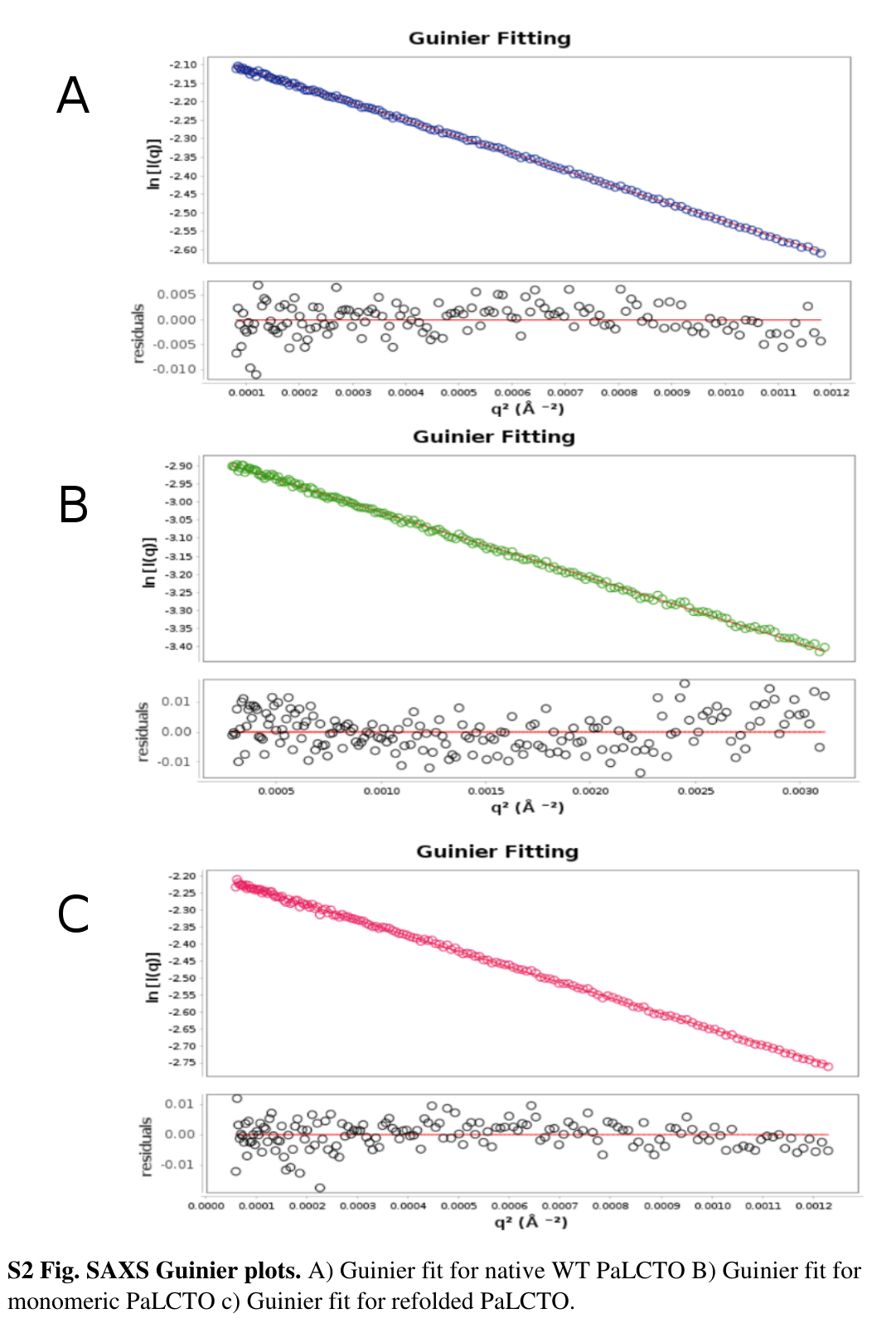

Supplement: S2 Fig — (PNG) [file pone.0223870.s002.png]

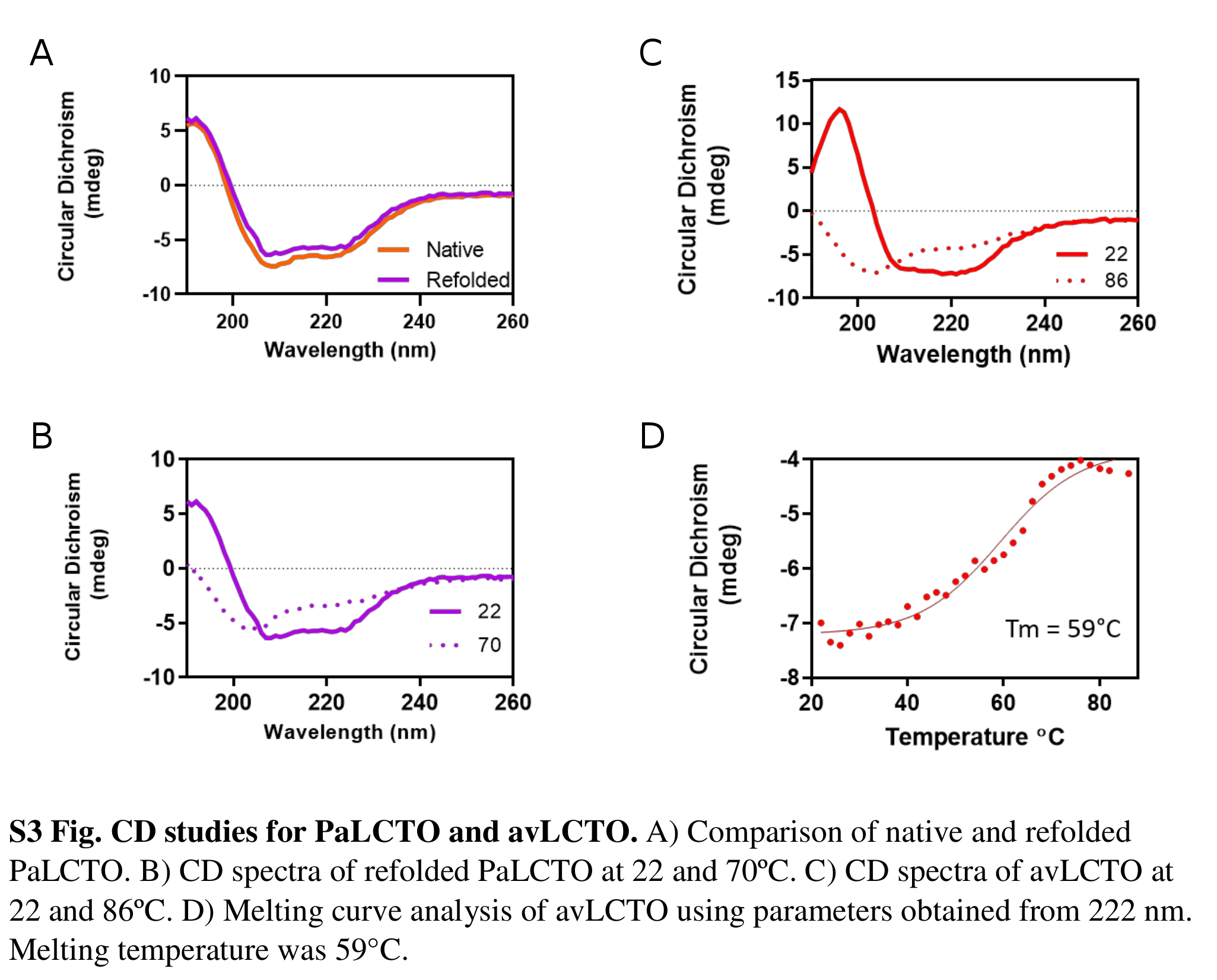

Supplement: S3 Fig — (PNG) [file pone.0223870.s003.png]

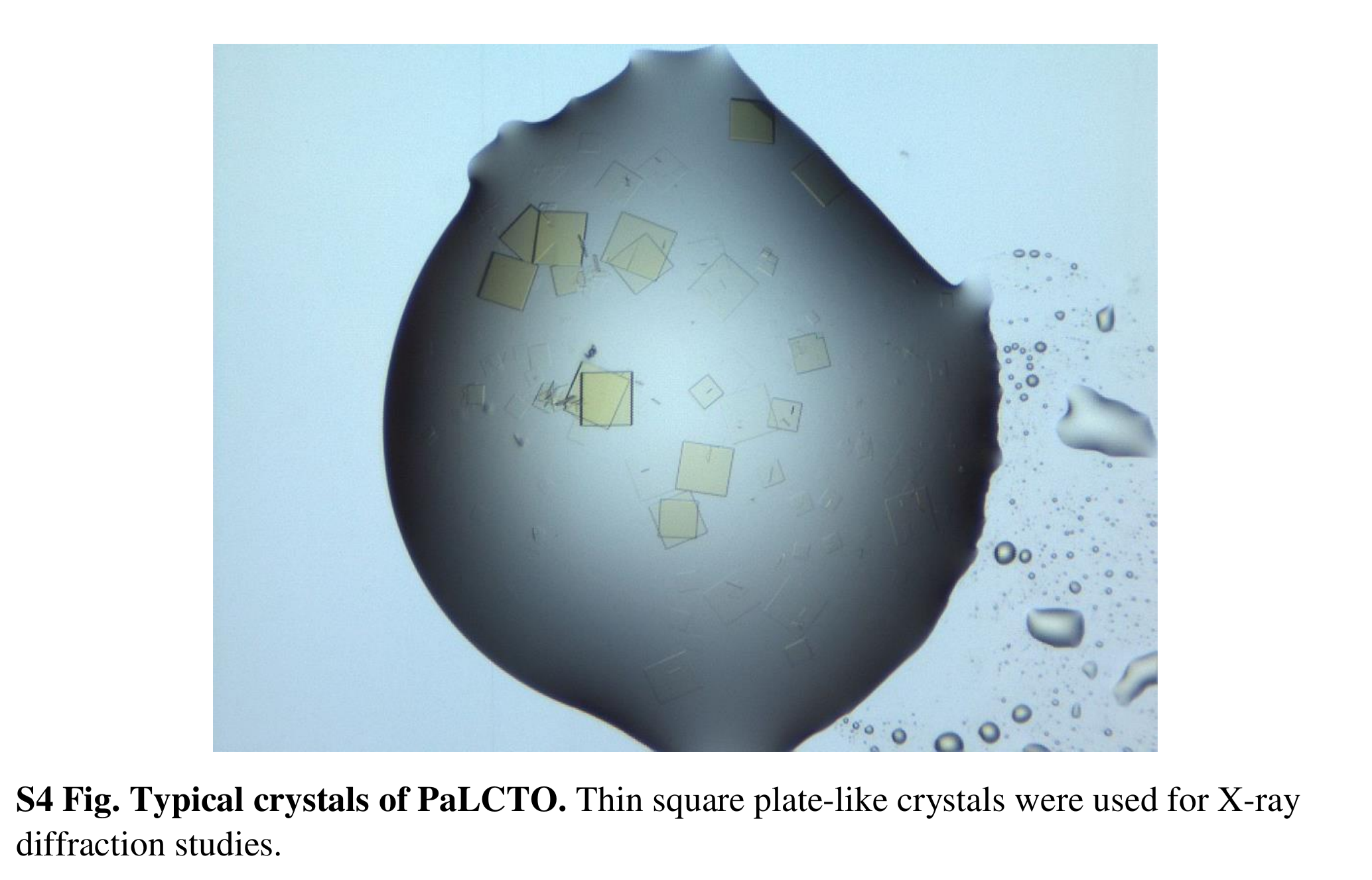

Supplement: S4 Fig — (PNG) [file pone.0223870.s004.png]

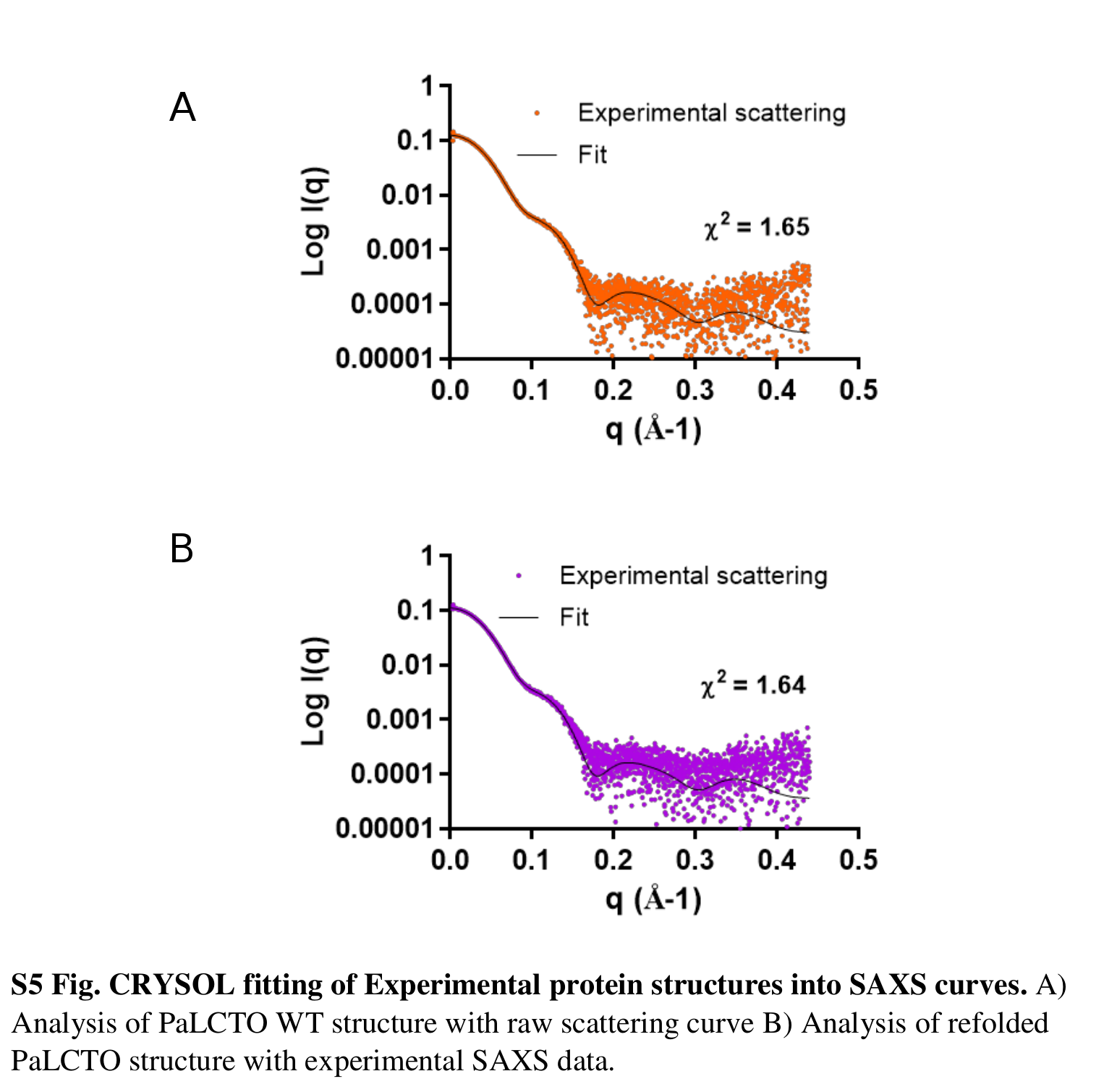

Supplement: S5 Fig — (PNG) [file pone.0223870.s005.png]

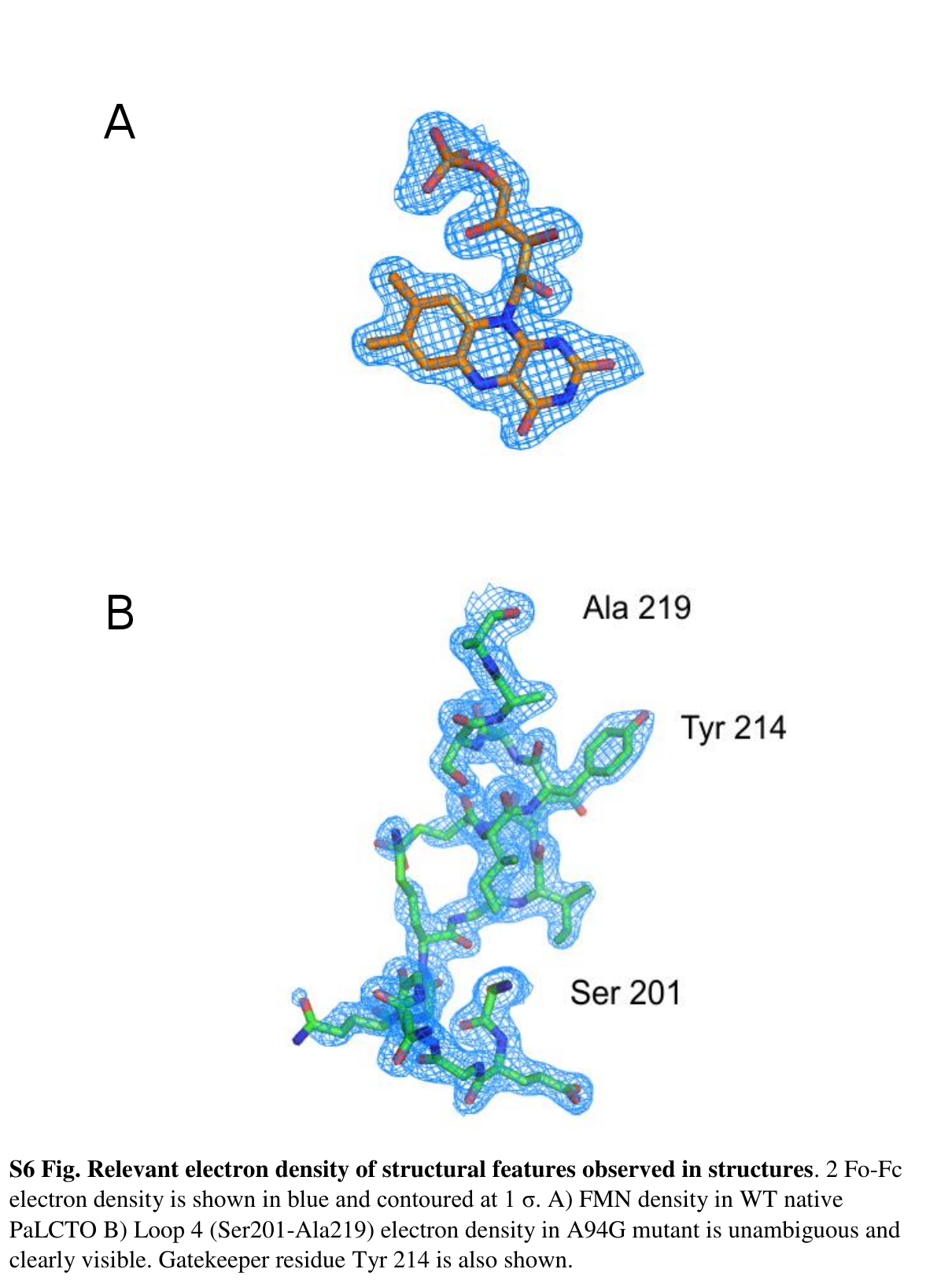

Supplement: S6 Fig — (PNG) [file pone.0223870.s006.png]

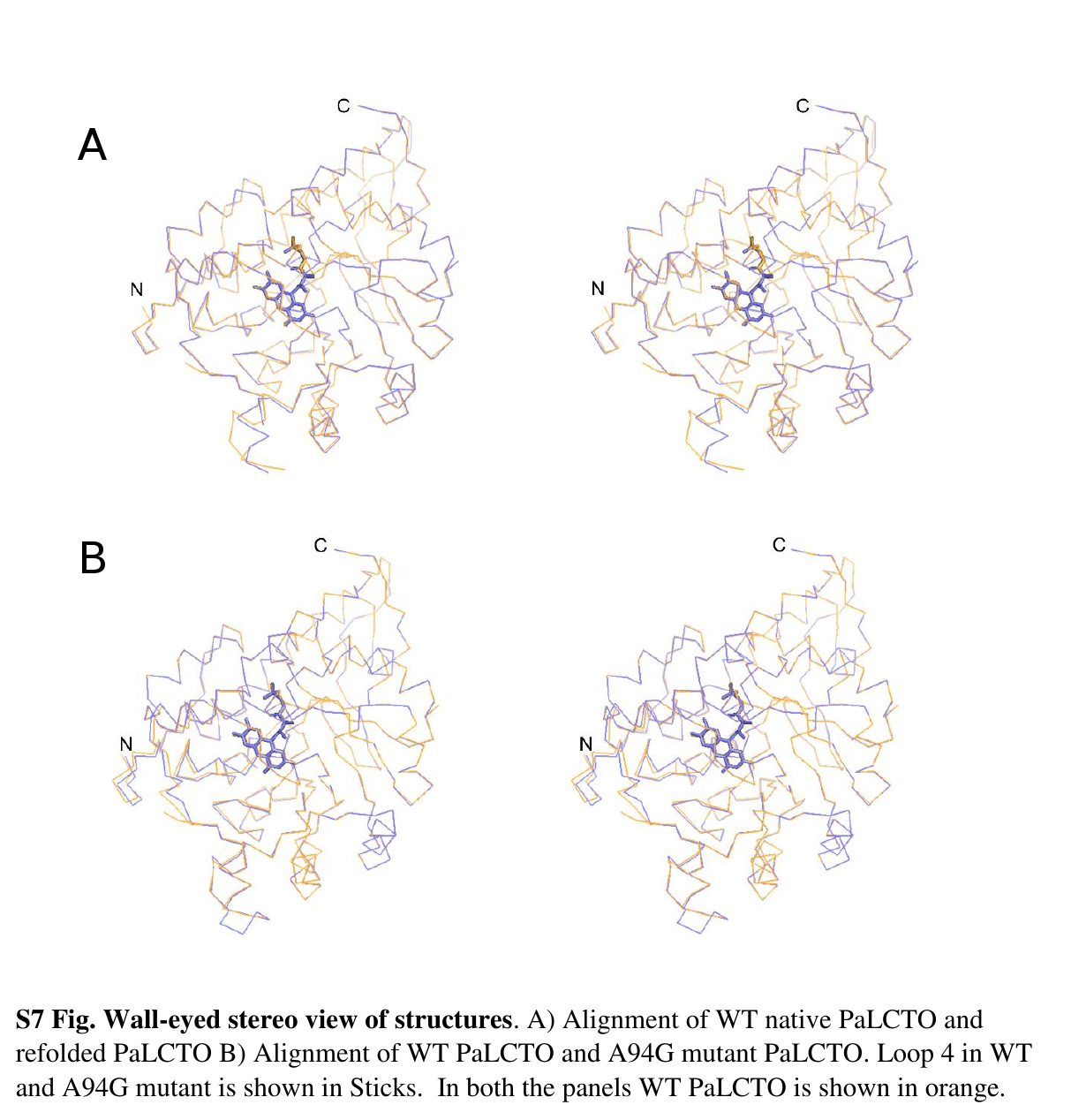

Supplement: S7 Fig — (PNG) [file pone.0223870.s007.png]

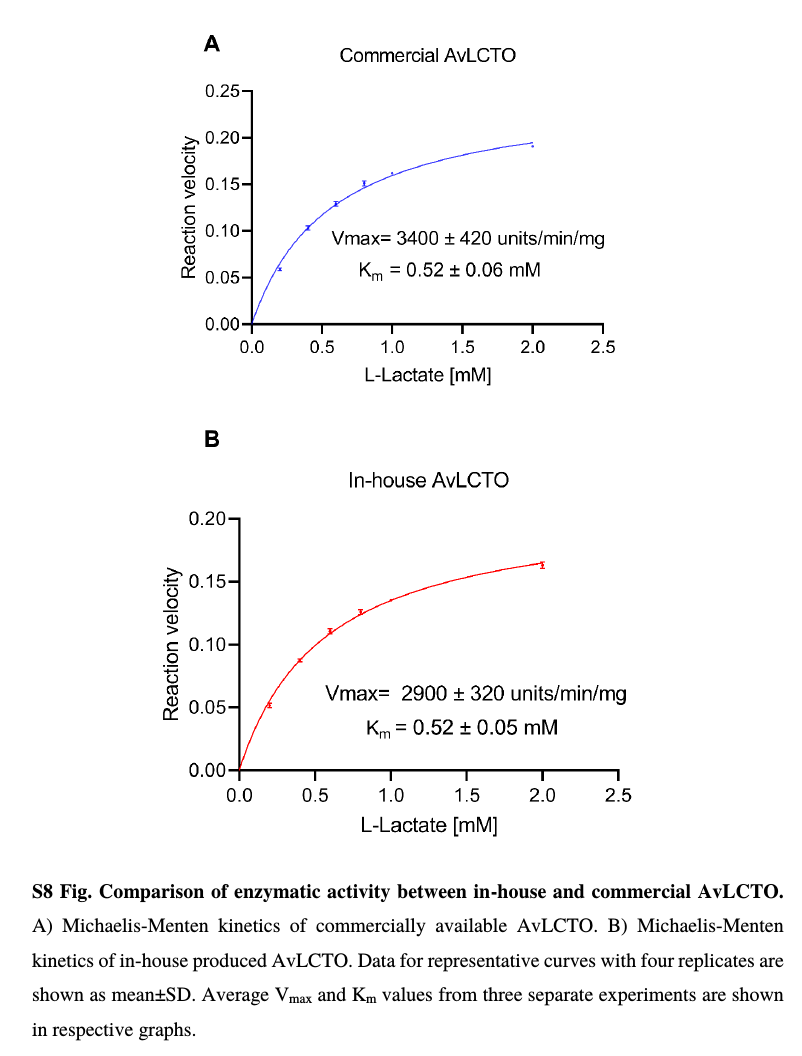

Supplement: S8 Fig — (PNG) [file pone.0223870.s008.png]

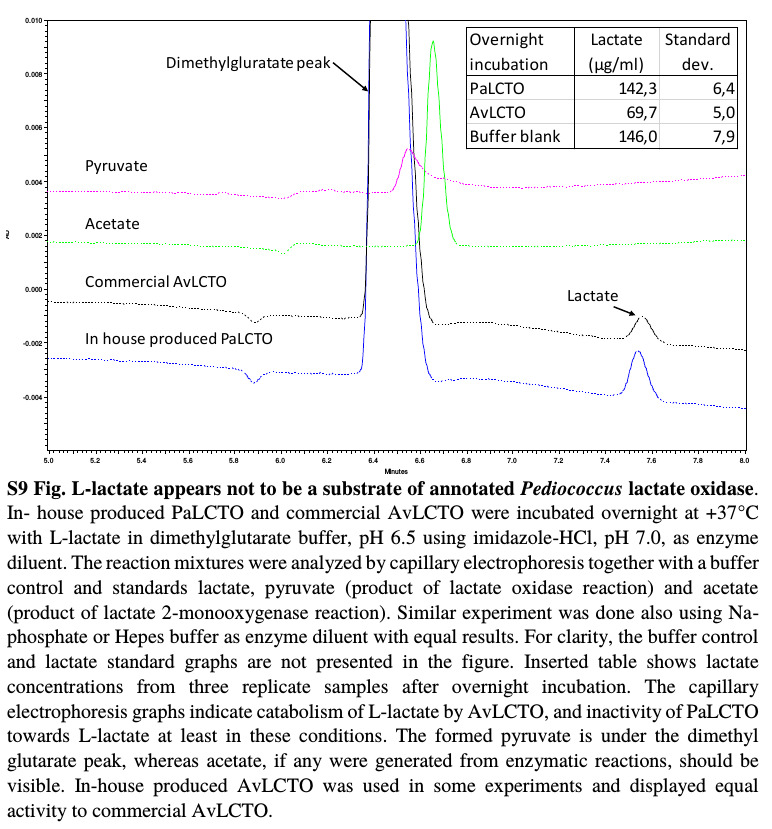

Supplement: S9 Fig — (PNG) [file pone.0223870.s009.png]
