# Supplementary material for: FMN-dependent oligomerization of putative lactate oxidase from Pediococcus acidilactici
Source: PLoS One. 2020 Feb 24;15(2):e0223870. doi: 10.1371/journal.pone.0223870 (PMC7039449; doi:10.1371/journal.pone.0223870)
Supplement: S1 Table — (PDF) [file pone.0223870.s010.pdf]

S1 Table. SAXS summary.

**Parameters / Data**			
**sample details**	**PaLCTO native WT**	**PaLCTO monomer**	**PaLCTO refolded WT**
Organism	*Pediococccus acidilactici*	*Pediococccus acidilactici*	*Pediococccus acidilactici*
Uniprot sequence IDs	E0NE46	E0NE46	E0NE46
Extinction coefficient (A280, 0.1% (w/v))	0.653	0.653	0.653
M from chemical composition (Da)	39678	39678	39678
SEC-SAXS column	Superose 6 increase 3.2/300	Superose 6 increase 3.2/300	Superose 6 increase 3.2/300
injected volume (μl)	60	60	60
Flowrate (ml/min)	0.075	0.060	0.075
Solvent (buffer)	30mM Hepes pH 7.5, 350 mM NaCl, 5% glycerol, 0.5mM TCEP	30mM Hepes pH 7.5, 350 mM NaCl, 5% glycerol, 0.5mM TCEP	30mM Hepes pH 7.5, 350 mM NaCl, 5% glycerol, 0.5mM TCEP
**SAXS data collection parameters**			
Beamline/Instrument /data processing	B21, DLS, UK	B21, DLS, UK	B21, DLS, UK
Beam size	250 x 250 μm	250 x 250 μm	250 x 250 μm
Detector	Eiger 4M	Eiger 4M	Eiger 4M
Wavelength (Å)	1	1	1
camera length (m)	4.014	4.014	4.014
q measurement range (per Å)	0.0032 to 0.38	0.0032 to 0.38	0.0032 to 0.38
Exposure time (sec)	3	3	3
sample configuration	SEC-SAXS with quartz cell capillary	SEC-SAXS with quartz cell capillary	SEC-SAXS with quartz cell capillary
sample temperature (^{o}C)	20	20	20
**software employed for SAXS data reduction, analysis, interpretation**			
SAXS data reduction	I(q) Vs q, solvent subtraction using Scatter	I(q) Vs q, solvent subtraction using Scatter	I(q) Vs q, solvent subtraction using Scatter
Extinction coefficient estimate	Protparam	Protparam	Protparam
Basic analysis : Guiner, p(r), Vp	PRIMUS from ATSAS, Scatter	PRIMUS from ATSAS, Scatter	PRIMUS from ATSAS, Scatter
Atomic structure modelling	CRYSOL	CRYSOL	CRYSOL
**structural parameters**			

**guinier analysis**			
I(0) cm^{-1}	0.1262	0.5779E-01	0.1113E+0
q range (Å-1)	0.0070 – 0.2174	0.0090 – 0.3450	0.0068 - 0.2170
R_g	36.7	23.2	36.8
qR_g max	1.27	1.26	1.28
**P(r) analysis**			
I(0) cm-1	0.1262 ± 0.4779E-04	0.5779E-01 ± 0.4591E-04	0.1113E+00 ± 0.5212E-04
Rg (Å)	0.3662 ± 0.1771E-01	0.2350E=02 ± 0.3780E-01	0.3673E+02 ± 0.1954E-01
dmax (Å)	113	85.3	113.14
q range (Å-1)	0.0070 - 0.2174	0.0090 – 0.3450	0.0068 – 0.2170
x^2 (total estimate from GNOM)	0.9505	0.71	0.9834
Porod volume, Po (Å-3)	271747	61973	271754
Molecular weight derived from -SAXS MoW2.0	153	40	151
**CRYSOL**			
x^2 (goodness of fit) with the atomic model	1.6	NA	1.6
predicted Rg (Å)	36.5	NA	36.62
vol (Å), Ra (Å), Dro e (Å-3)	186186	NA	197197
